# Synthesis and characterization of multiwalled CNT–PAN based composite carbon nanofibers via electrospinning

**DOI:** 10.1186/s40064-016-2051-6

**Published:** 2016-04-19

**Authors:** Narinder Kaur, Vipin Kumar, Sanjay R. Dhakate

**Affiliations:** Department of Physics, Krishna Institute of Engineering and Technology, Ghaziabad, India; National Physical Laboratory, New Delhi, India

**Keywords:** Polyacrylonitrile, Nanocyl multiwalled carbon nanotubes

## Abstract

Electrospun fibrous membranes find place in diverse applications like sensors, filters, fuel cell membranes, scaffolds for tissue engineering, organic electronics etc. The objectives of present work are to electrospun polyacrylonitrile (PAN) nanofibers and PAN–CNT nanocomposite nanofibers and convert into carbon nanofiber and carbon-CNT composite nanofiber. The work was divided into two parts, development of nanofibers and composite nanofiber. The PAN nanofibers were produced from 9 wt% PAN solution by electrospinning technique. In another case PAN–CNT composite nanofibers were developed from different concentrations of MWCNTs (1–3 wt%) in 9 wt% PAN solution by electrospinning. Both types of nanofibers were undergone through oxidation, stabilization, carbonization and graphitization. At each stage of processing of carbon and carbon-CNT composite nanofibers were characterized by SEM, AFM, TGA and XRD. It was observed that diameter of nanofiber varies with processing parameters such as applied voltage tip to collector distance, flow rate of solution and polymer concentrations etc. while in case of PAN–CNT composite nanofiber diameter decreases with increasing concentration of CNT in PAN solution. Also with stabilization, carbonization and graphitization diameter of nanofiber decreases. SEM images shows that the minimum fiber diameter in case of 3 wt% of CNT solution because as viscosity increases it reduces the phase separation of PAN and solvent and as a consequence increases in the fiber diameter. AFM images shows that surface of film is irregular which give idea about mat type orientation of fibers. XRD results show that degree of graphitization increases on increasing CNT concentration because of additional stresses exerting on the nanofiber surface in the immediate vicinity of CNTs. TGA results shows wt loss decreases as CNT concentration increases in fibers.

## Background

Electrospinning is a most efficient technique to generate fibers with submicron diameters. Electrospun nanofibers mats are more promising because of high surface area and porosity, which have applications in air filtration, tissue engineering, drug delivery, and energy storage materials (Doshi and Reneker 1995; Formhals 1944). Reliable production of porous nanofibers in a simple and inexpensive way has been attempted by number of groups (Xia and Li 2006) showed that, by using a coaxial spinneret with miscible solvents and immiscible polymers, highly porous fibers could be obtained (Xia and Li 2006). Since the beginning of this century, researchers all over the world have been re-looking at a century old process (Cooley and Morton 1902) currently it is known as electrospinning (Hagewood 2004). Probably unknown to most researchers for most of the last century, electrospinning is able to produce continuous fibers from the submicron diameter down to the nanometer diameter. It was not until the mid-1990s with interest in the field of nanoscience and nanotechnology that researchers started to realize the huge potential of the process in nanofiber production (Doshi and Reneker 1995). Nanofibers and nanowires with their huge surface area to volume ratio, about a thousand times higher than that of a human hair, have aspect area.

Carbon fibers are manufactured through heating and stretching treatments (Shenoy et al. 2005). Polyacrylonitrile (PAN) and pitch are the two most common raw products used to produce carbon fibers. PAN is a synthetic fiber that is pre manufactured and wound onto spools, and pitch is a coal-tar petroleum product that is melted, spun, and stretched into fibers. The production of carbon nano fibrils will achieve a unique property (Mechanical, Electrical and Physical) by keeping the same amount of crystallite size from core to skin and increasing the surface area per unit volume which mimic the same structure of MWCNT (Saito et al. 1998).

This paper reports our findings on testing this concept by electrospinning mixtures of Chopped PAN co-polymer micro fibers of diameter 12.5 µm having polyacrylonitrile with a 6 % monomer methyl methacrylate and *N*,*N*-dimethylformamide (DMF) of 99 % purity (B. Pt. = 157 °C) and also Functionalized Carbon Nanotubes (CNT) are used as fillers (Haddon and Itkis 2008). Fibers uniformity and diameter (75–1500 nm) have been shown to increase with increasing concentrations of CNT by controlling solution and processing parameters in whole study (Haddon and Itkis 2008).

## Experimental details

### Materials

#### **Polymer**

 Chopped PAN co-polymer micro fibers of diameter 12.5 µm having polyacrylonitrile with a 6 % monomer methyl methacrylate was used as source of PAN. Molecular weight of PAN is 53.0626 ± 0.0028 g/mol, C 67.91 %, H 5.7 %, N 26.4 %.

#### **Solvent**

*N*,*N*-dimethylformamide (DMF) of 99 % purity (B. Pt. = 157 °C) from Fisher Scientific.

#### **Filler**

 Functionalized Carbon Nanotubes (CNT) from Nanocyl.

### Preparation of PAN nanofibers

The 9 wt% PAN copolymer solution was prepared by taking 0.9 g chopped PAN microfibers was socked in 9.1 g of DMF and mix with glass rod. The mix was sonicated in ultrasonicator till it gives a clear solution and stirred continuously on magnetic stirrer for 5 h to mix well the contents.

### Preparation of PAN–CNT composite nanofibers

PAN–CNT (1–3 wt%) 9 % solutions were prepared in following steps: at first 0.01 g functionalized CNTs were dispersed in *N*,*N*-dimethylformamide. The mixture was kept in sonicator for 5–8 h to break bundles of CNTs and magnetic stirring to disperse well CNTs in DMF. A good dispersion was achieved in 7 h. Calculated quantity of PAN copolymer chopped microfibers were added in dispersion solution and kept on magnetic stirrer for overnight to mix well the polymer in CNT dispersion. Solution was sonicated for 1 h and then electrospun with ESPIN instrument at voltage 15 kV, flow rate 0.2 ml/h, drum speed 2000 rpm, distance 15 cm to produce PAN–CNT composite nanofibers. It was difficult to prepare solution using higher concentration of CNT. The problem faced was that, in high quantity of CNTs polymer not get dispersed well and form a solid mass. So both CNTs and polymer were dispersed in DMF separately. CNTs and DMF mixture was sonicated for 1 h till all bundles breakup and then kept on magnetic stirrer, good dispersion observed in sun light. Polymer was soaked in DMF well and sonicated for 1 h. Then it was kept on magnetic stirrer till a clear solution was obtained. Same procedure was carried out for PAN–CNT concentrations (2–3 wt%).

Both types of nanofibers were undergone through oxidation, stabilization, carbonization and graphitization. At each stage of processing of carbon and carbon-CNT composite nanofibers were characterized by SEM, AFM, XRD and TGA.

## Results and discussions

### SEM analysis

**Fig. 1:**
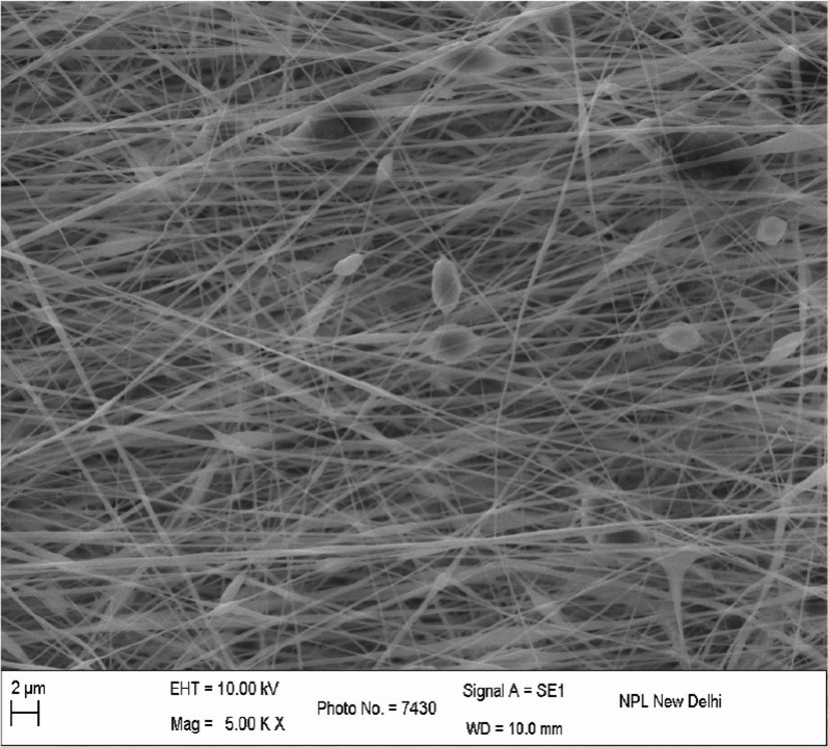
SEM image electrospun PAN copolymer nanofibers with 9 wt% 1000 rpm, flow rate 0.2 ml/h, applied voltage 15 kV, 10 cm

**Fig. 2:**
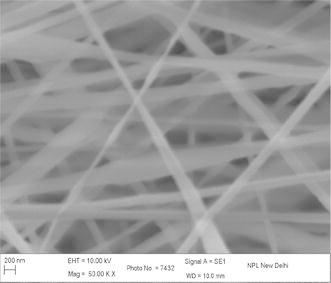
SEM image PAN–CNT nanocomposite with 1 % MWCNTs

**Fig. 3:**
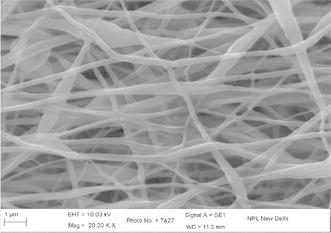
SEM images of electrospun PAN–CNT stabilized nanofibers at 310 °C with 3 wt% of CNT and drum speed 2000 rpm, distance 10 cm, flow rate 0.3 ml/h, voltage 15 kV

**Fig. 4:**
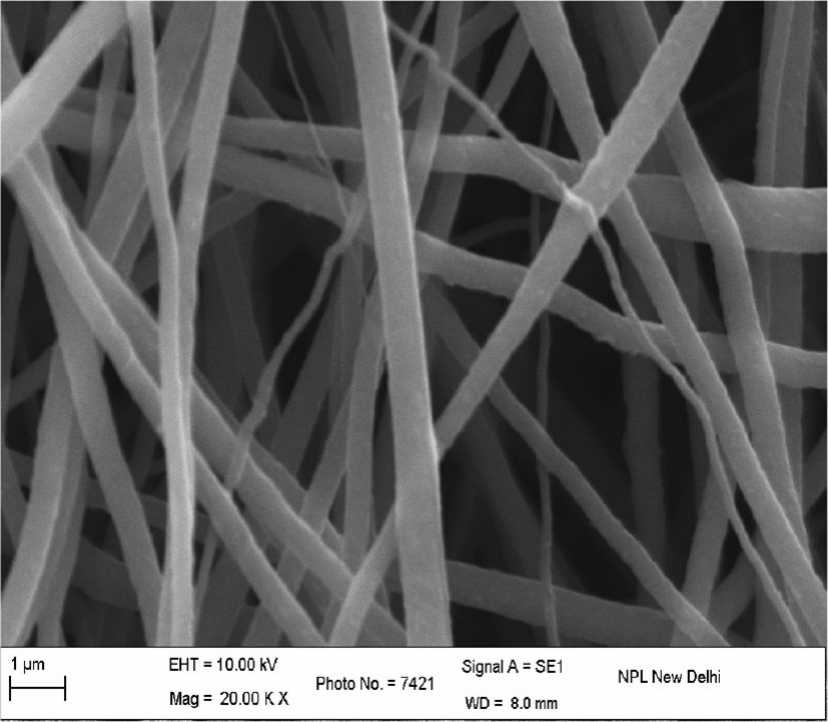
SEM images of electrospun PAN–CNT carbonized nanofibers at 1000 °C with 3 wt% of CNT and drum speed 2000 rpm, distance 10 cm, flow rate 0.3 ml/h, voltage 15 kV

The concentration, viscosity, and conductivity of the solution as well as the applied voltage and distance between the charged electrode and the grounded target were adjusted in order to obtain stable electrospinning jet (Drozin 1955). Figure 1 shows the micrograph of electrospun nanofibers (Gupta and Wilkes 2003) prepared from solution of 9 wt% of PAN concentration with flow rate 0.2 ml/h and collector speed 1000 rpm, applied voltage 15 kV in which tip to collector distance varies from 10 to 20 cm at EHT = 10 kV and at different magnification. It is observed that with increasing the tip to collector distance, fiber diameter decreases. At tip to collector distance 10 cm, fiber diameter is in the range of 100–175 nm and decreases to 75–175 nm on increasing distance to 15 cm. In this case the variation is comparatively less as compared to tip to collector distance 10 cm. This variation in diameter is due to the instability in jet due to change in repulsive forces (Baumgarten 1971). Figure 2 shows the SEM image of PAN–CNT composite with 1 % MWCNTs. The CNTs are aligned in nanocomposite, which is distinguished by color of PAN nanofibers. The presence of black color in fiber, it indicates the presence of CNTs in nanofibers. Since the MWNTs possess a high electron density compared with the PAN polymer matrix, the nanotubes appear as darker tubular structures embedded in the PAN distorted nanofibers with black impression. The stabilization is one of the important steps and play important role in controlling the properties of final fibers (Ramakrishna et al. 2005; Ko et al. 2003; Khil et al. 2005). Figure 3 shows the stabilized PAN–CNT 3 wt% nanofibers. The oxidative stabilization was carried out at 310 °C and kept isothermally for 1 h. It is found that, on stabilization fibers diameter decreases as compared as spun nanofiber diameter. Figure 4 shows the SEM image of carbonized fiber with 3 wt% of CNTs incorporated during the processing. From the figure it is observed that there visible difference in the morphology of carbon-CNT composite nanofibers. At higher content of CNTs, CNTs are might be not individually aligned during electrospinning. Therefore, from the surface of carbon-CNTs nanofiber, it is visible bundles (He et al. 2004) in the range of 50–100 nm in the fiber of diameter 300–400 nm (Guo et al. 1955).

### AFM analysis

**Fig. 5:**
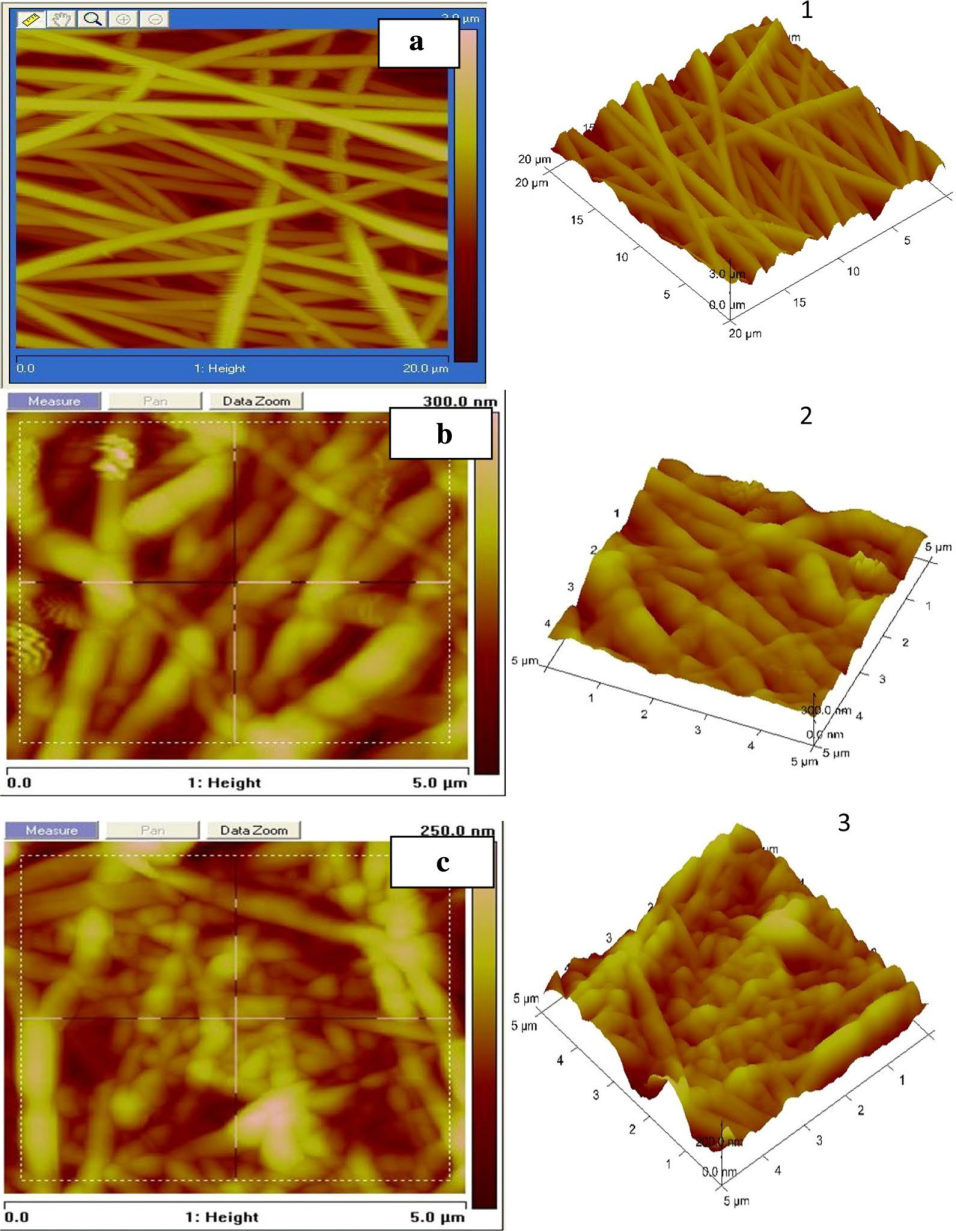
2D & 3D AFM images of PAN copolymer nanofibers mat of concentration 9 wt% in DMF. **a**(**1**) nanofibers, **b**(**2**) stabilized, **c**(**3**) carbonized

**Fig. 6:**
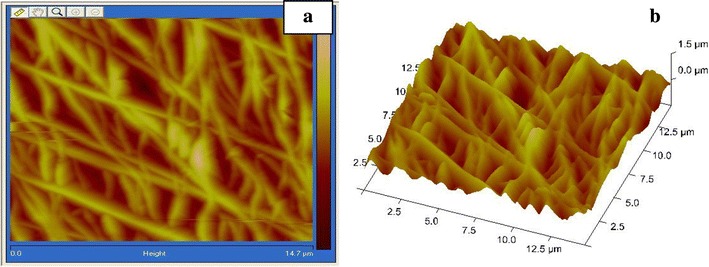
AFM images of PAN–CNT carbonized from concentration 3 wt% of CNT, tip to collector distance 10 cm, drum speed 2000 rpm, **a** 2D image, **b** 3D image

Figure 5 shows 2D & 3D AFM images of PAN copolymer nanofibers mat of over an area 5 µm × 5 µm of concentration 9 wt% in DMF for nanofiber, stabilized nanofiber and carbonized nanofiber. This shows that nanofibers are randomly oriented and surface roughness of film is 32.3 nm. The surface area of film is 23 µm^2^ and from bar analysis of surface of film, nanofibers have diameter in the range 75–100 nm & depth of film is 273 nm (Ramakrishna et al. 2005; Samatham et al. 2006). But on carbonization, surface area decreases to 22.4 µm^2^ and surface roughness to 27.0 nm. The depth of film also decreases to 268 nm and fiber diameter to 70–90 nm. The change in depth and diameter is related to shrinkage of fiber film due to cyclization reactions take place at higher temperature. Figure 6 shows the AFM images of PAN–CNT 3 wt% carbonized fibers shows two kinds of regions, lighter and darker. The lighter regions are related to the highest points, and the darker regions are related to pores and valleys. This suggests that surface of film become more asymmetric on carbonization sharp spike in the picture is due to non orientation of fibre surface in one plane. It shows that surface of film is irregular which give idea about mat type orientation of fibers and high percentage of CNT was easily seen from fibre sheet which has darker black color. Diameter of fibre decreases due to presence of embedded CNT’s because diameter of Taylor cone is reduced due to high electron density. In carbonization there occur the change in depth and diameter due to shrinkage of fiber film due to cyclization reactions take place at higher temperature (Ko et al. 2003; Hendricks et al. 1964; Taylor 1969).

### XRD analysis

**Fig. 7:**
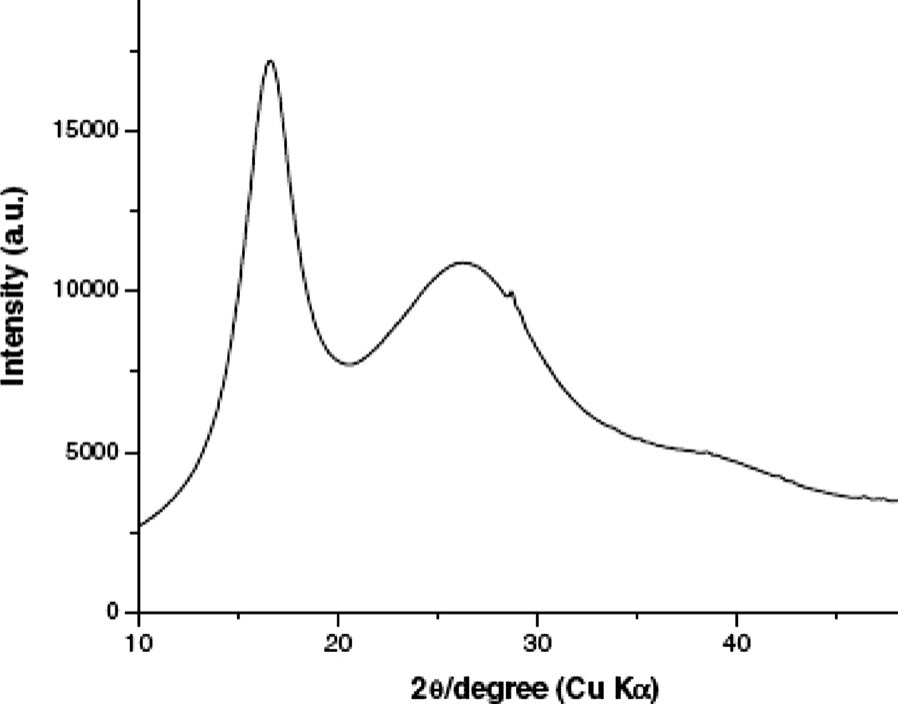
XRD spectra of PAN nanofibers

**Fig. 8:**
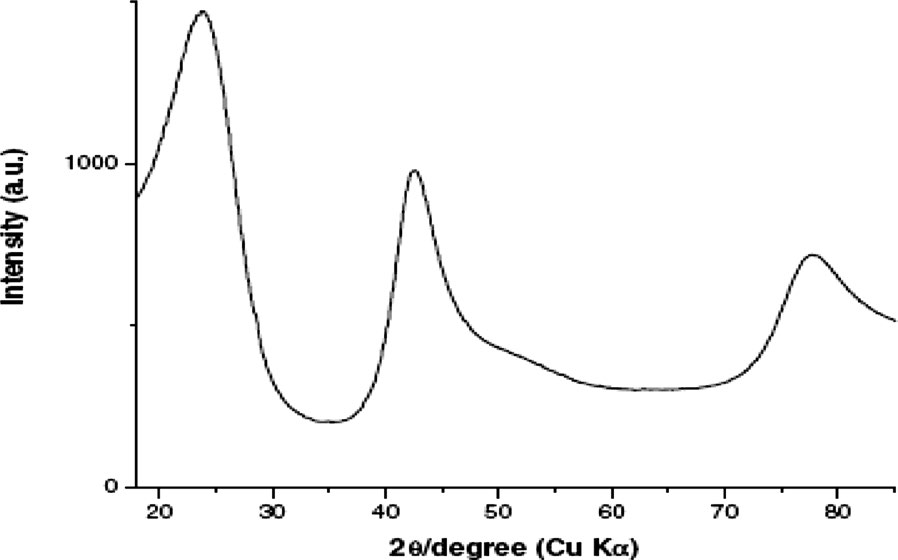
XRD spectra of carbon nanofiber

Figures 7 and 8 shows XRD patterns of PAN nanofibers and carbon nanofibers. PAN fiber shows strong diffraction peak centered around 2θ angle of 16.67° and 29.462°, these two peaks represents the X-ray reflection of the (110) of a hexagonal structure and (112) crystallographic planes in PAN. However, in case of the PAN–CNT 1 wt% nanofibers, curve “B”, the peak centered on 2θ angle of 16.67° and 29.462°, these two peaks represents the X-ray reflection of the (110) of a hexagonal structure and (112), and 2θ angle of 44.37° represents the X-ray reflection of the (004) this is due to presence of CNT. In case of the PAN–CNT 2–3 wt% nanofibers, curve “C”, the peak centered on 2θ angle of 16.67° and 29.462°, these two peaks represents the X-ray reflection of the (110) of a hexagonal structure and (112), and 2θ angle of 44.37° represents the X-ray reflection of the (004) this is due to presence of CNT. But intensity of peaks is very high as comparison to 1 wt% of CNT (Hagewood 2004). In carbonized nanofibers intense diffraction peak as compared to stabilize fibres around 2θ angle of 24°–26° is attributed to (002) crystallographic plane of graphite crystallite. Degree of graphitization is determined from XRD results (Kim and Reneker 1999) by using formula (g = ((0.3440-d_200_)/(0.3440–0.3354)) × 100 and d_200_ = nλ/2sinθ where λ is wavelength, θ is diffraction angle and 0.3440 is interlayer spacing of fully non graphitized carbon (nm). Degree of graphitization for PAN nanofibers is −20 % and for PAN–CNT nanofibers is −41.86. Degree of graphitization increases due to presence of CNT in fibers.

### TGA analysis

**Fig. 9:**
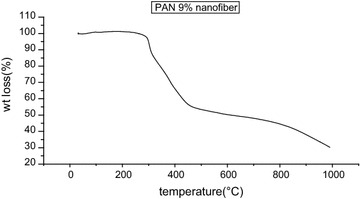
TGA graph of PAN nanofibers from concentration 9 wt%, drum speed 2000 rpm, tip to collector distance 10 cm

**Fig. 10:**
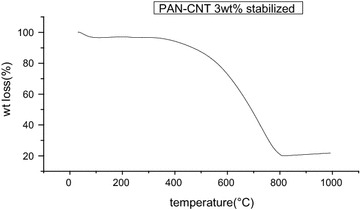
TGA graph of PAN–CNT stabilized with concentration of CNT 3 wt%, drum speed 2000 rpm, tip to collector distance 15 cm

Mettle Toledo TGA star system was used for TGA analysis up to 1000 °C temp and rate of 10°/c in the nitrogen atmosphere at flow rat 10 ml/min. Figure 9 shows that in case of 9 wt% concentration PAN copolymer nanofibers after heating at 1000 °C residue remaining found to be 68.158 % i.e. wt loss was 31.842 %. The wt loss in nanofibers is very less which shows stability increase in nano size of fibers. This may be due to the high surface area of nanofibers which provide stability as well as complete stabilization reaction. Figure 10 shows that PAN–CNT stabilized nanofibers after heating at 310 °C residue remaining found to be 21.8283 % i.e. wt loss was 78.1717 %. This wt loss is more as in case of PAN–CNT stabilized. This may be again due to the decrease in fiber size because as concentration of CNT increases, fiber diameter also decrease and surface area increase. This may be due to the high surface area of nanofibers which provide stability as well as complete stabilization reaction.

**Fig. 11:**
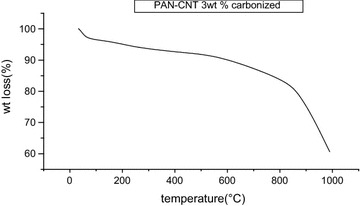
TGA graph of PAN–CNT carbonized with concentration of CNT 3 wt%, drum speed 2000 rpm, tip to collector distance 15 cm

Figure 11 shows that PAN–CNT carbonized nanofibers after heating at 310 °C residue remaining found to be 60.8283 % i.e. wt loss was 39.1717 % (Yarin et al. 2001). This wt loss is less in case of PAN–CNT carbonized as comparison to PAN–CNT stabilized because of increase in stability in structure (Teraoka 2002; Huang et al. 2003), and removal of nitrogen, water and hydrogen. HCN and another gases and also carbon content increases on carbonization (Zhou and Liu 2010).

## Conclusions

In the present study we have successfully used the electrospinning for the synthesis of PAN–CNT carbon nanofibers. SEM images shows that the fiber diameter varies from 75 to 1500 nm on increasing concentration of CNT. The presence of black color in fiber, it indicates the presence of CNTs in nanofibers. AFM images show that the surface morphology of the composite nanofibers is smooth at lower concentration of MWCNT but rough at high concentration of MWNTs. Since the MWNTs possess a high electron density compared with the PAN polymer matrix, the nanotubes appear as darker tubular structures embedded in the PAN nanofibers. It is also seen that on Stabilization fibers diameter decreases due to shrinkage in fibers. Degree of graphitization for PAN nanofibers is −20 % and for PAN–CNT nanofibers is −41.86. Degree of graphitization increases due to presence of CNT in fibers. TGA results shows that wt loss is small in case of PAN nanofibers because of high surface area of nanofibers while in case of carbonized PAN–MWCNT carbon nanofibers wt loss is small as comparison to PAN–CNT stabilized because of increase in stability in structure, and removal of nitrogen, water and hydrogen. HCN and another gases and also carbon content increases on carbonization.
